# Ingenuity of minimally invasive thoracoscopic lobectomy for undiagnosed pulmonary tumour

**DOI:** 10.1002/rcr2.657

**Published:** 2020-09-03

**Authors:** Sumitaka Yamanaka, Takashi Yoshimatsu, Takeaki Miyata, Hanae Higa

**Affiliations:** ^1^ Department of Thoracic Surgery Tokyo Shinagawa Hospital Tokyo Japan; ^2^ Department of Thoracic Surgery Fukuoka Wajiro Hospital Fukuoka Japan; ^3^ Department of Thoracic Surgery Shinkuki General Hospital Kuki Japan

**Keywords:** Intercostal nerve, neuralgia, reduced port surgery, tuberculosis, video‐assisted thoracic surgery

## Abstract

A 51‐year‐old man was referred to our hospital, with a dumbbell‐shaped nodule measuring 40 mm in the right upper lobe of the lung. He was a current smoker with diabetes mellitus and bronchial asthma. The transbronchial biopsy was performed. However, definitive diagnosis was not obtained from the excised specimens. Bacterial culture of bronchial lavage fluid also yielded negative results, including for tuberculosis. After eight months of observation, the tumour had slightly increased in size. Surgery was planned to resect the tumour for definitive diagnosis. Because of the size of the tumour, a lobectomy of the lung was scheduled with the patient's consent. Four small incisions, each less than 1.2 cm long, were made in the chest wall for thoracoscopic surgery. To remove the specimen, we made a 3‐cm longitudinal incision 1 cm below the xiphisternal joint. The patient complained of no chest pain after surgery. The post‐operative course was uneventful.

## Graphical abstract

This study describes video‐assisted thoracic surgery (VATS) with minimal chest incisions to prevent chronic post‐operative intercostal neuralgia and paraesthesia, using a small subxiphoid incision only to remove the specimen.

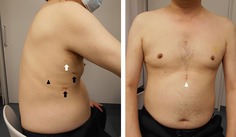

## Introduction

Multiportal video‐assisted thoracic surgery (MVATS) has already served as the first‐line operative procedure for lung diseases [1], and uniportal video‐assisted thoracic surgery (UVATS) has since emerged as a vital alternative to MVATS, offering the advantages of better cosmetic outcomes and improved post‐operative results for patients [2]. However, in association with chronic complication after thoracic surgery, a prospective study reported no difference in the incidence or severity of chronic pain at six months post‐operatively in patients who underwent thoracotomy or thoracoscopy [3].

We report the method for video‐assisted thoracic surgery (VATS) with minimal chest incisions to prevent chronic post‐operative intercostal neuralgia and paraesthesia, using a small subxiphoid incision only to remove the specimen, for the benign lung tumours that unavoidably require more than a wedge resection.

## Case Report

**Figure 1:**
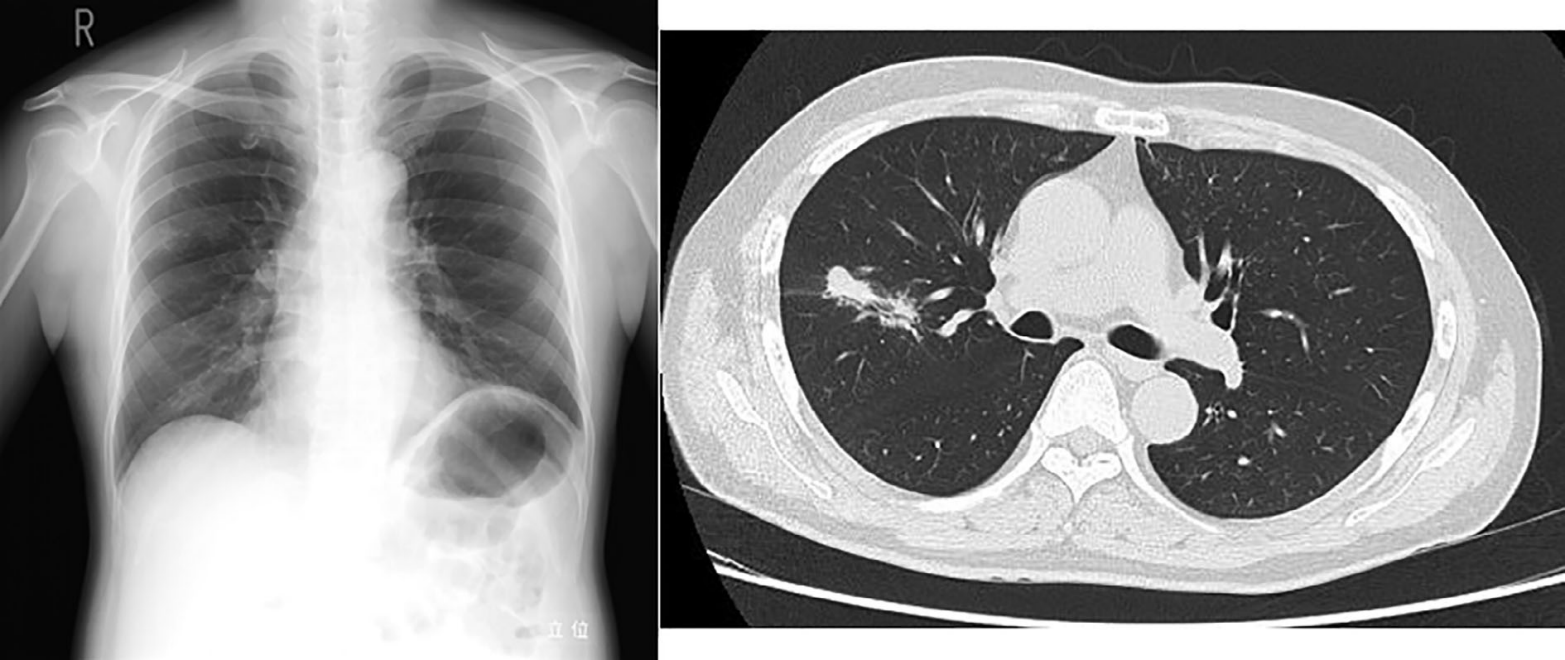
Chest X‐ray and computed tomography before operation. An abnormal infiltrative shadow is indicated in the right upper lung field on the chest X‐ray. A dumbbell‐shaped nodule measuring 40 mm × 10 mm is seen in the right upper lobe on the chest computed tomography.

A 51‐year‐old man was referred to our hospital, with a dumbbell‐shaped nodule measuring 40 mm in the right upper lobe of the lung (Fig. 1). He was a current smoker and also had hypertension, diabetes mellitus, and bronchial asthma. Imaging findings suggested the possibility of a lung cancer, so transbronchial biopsy was performed. However, definitive diagnosis was not able to be obtained from the biopsy specimens. Bacterial culture of bronchial lavage fluid also yielded negative findings, including for tuberculosis. After eight months of observation, computed tomography showed that the tumour had slightly increased in size. Surgery was therefore planned to resect the tumour and reach a definitive diagnosis. Because of the size of the tumour, a lobectomy of the lung was scheduled with the patient's consent.

The patient was placed in the left lateral decubitus position under general anaesthesia. We then made a 1.2‐cm skin incision for insertion of a flexible thoracoscope and endoscopic autosuturing devices in the seventh intercostal space along the midaxillary line. We used a surgical scalpel only for cutting the dermis, and then gently separated muscles with forceps to access the thoracic cavity. Next, we made a 1.2‐cm skin incision for the main port in the fourth intercostal space along the anterior axillary line for endoscopic forceps and a vessel‐sealing device. In addition, a 5.5‐mm skin incision in the third intercostal space along midaxillary line and a 3.5‐mm skin incision in the fifth intercostal space along the posterior axillary line were placed to achieve effective visual overview of the surgical site.

The surgical procedure was basically the same as for conventional MVATS (Video S1, Supporting Information). We completed a typical right upper lobectomy and lymph node dissection. The lobectomy took approximately 3 h and there was a small amount of bleeding.

To remove the specimen, we created a 3‐cm longitudinal incision 1 cm below the xiphisternal joint. We subsequently incised the linea alba, and bluntly reached the right chest cavity using the index finger. The specimen was placed inside a tissue bag and removed through the subxiphoid incision (Video S2, Supporting Information). An intercostal nerve block with ropivacaine hydrochloride hydrate was performed at the end of the surgery.

Intravenous patient‐controlled analgesia (fentanyl citrate) was used for 18 h post‐operatively instead of epidural analgesia. Numerical rating pain scale (NRS) at rest has consistently been 1/10 since 1 h post‐operatively. The patient followed an uneventful post‐operative course. The chest drain was removed 18 h post‐operatively. He was discharged without any wound complications (NRS, 0/10).

**Figure 2:**
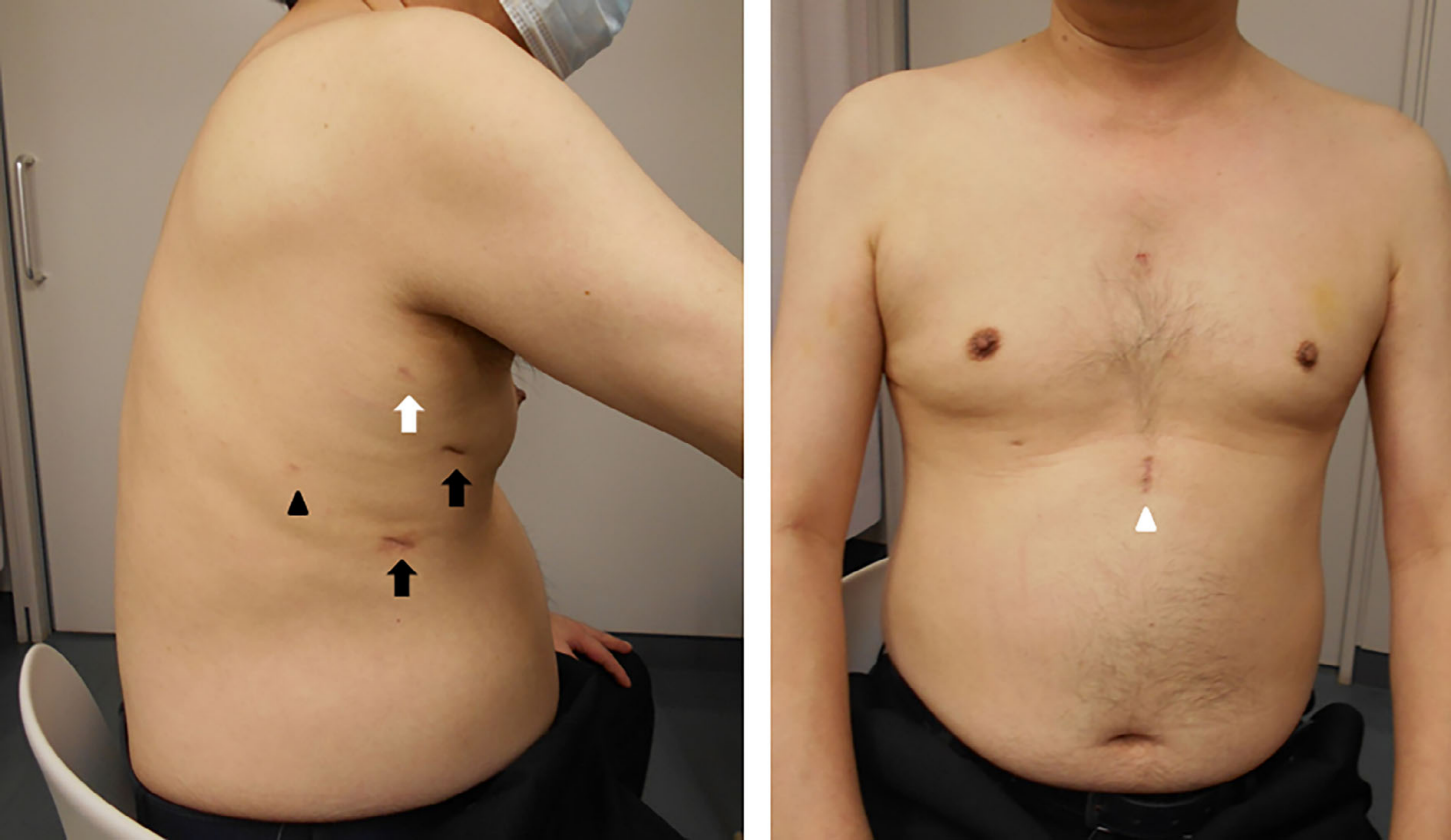
Operative scars six months after operation. Operative scars are clear and the patient did not complain of any pain, neuralgia, or paraesthesia of the chest wall. Wounds are 1.2 cm (black arrow), 5.5 mm (white arrow), 3.5 mm (black arrowhead), and 2.5 cm (white arrowhead).

The pathological diagnosis was tuberculoma, and antibacterial drugs were administered. The patient has not complained of any neuralgia or paraesthesia (NRS, 0/10) at the time of writing, six months post‐operatively (Fig. 2).

## Discussion

The advent of VATS has ameliorated post‐operative Quality of life for patients. Since its introduction, MVATS has become the first‐line procedure for anatomical lobectomies of lung neoplasms [1]. UVATS has recently emerged as a viable alternative to MVATS. In addition, subxiphoid UVATS has been recently reported as a surgery that does not risk damage to the intercostal neurovascular band giving rise to neuralgia, but most reports have only described the feasibility, safety, and perioperative outcomes [4].

The intercostal nerves give rise to the lateral cutaneous branch anterior to the midaxillary line, which sends subcutaneous fibres anteriorly and posteriorly. The rectus abdominis muscle originates from the costal cartilages or fifth to seventh ribs, and is innervated by anterior branches from the seventh through 12th intercostal nerves [5]. Subcostal incision can avoid direct damage to the intercostal bundle running in the costal groove, but can also impair the abdominal muscle directly and peripheral branches of the intercostal nerves innervating the abdominal muscles, which might consequently result in rectus abdominis muscle atrophy as a chronic adverse effect [5]. In addition, subxiphoid UVATS is generally difficult for cases with obesity or cardiac hypertrophy. For the above‐mentioned reasons, we have not usually adopted subxiphoid VATS.

The prospective study reported no difference in the incidence or severity of chronic pain at six months post‐operatively in patients who underwent thoracotomy or thoracoscopy [3]. Even small incisions in the chest wall of around 4 cm in diameter for UVATS or MVATS might still cut peripheral muscular and/or skin branches of the intercostal nerves. This is the reason that we did not choose UVATS and, alternatively, tried to minimize chest wall skin incisions and to attempt careful, minimum blunt dissection of the chest wall muscles to place access ports into the thoracic cavity without electrocautery. Although no post‐operative complications have been observed to date, careful long‐term observation remains crucial for novel surgical methods.

The modified reduced port size VATS with a small subxiphoid incision provides sufficient operative views to perform surgery safely, achieving cosmetic benefits and improving post‐operative quality of life for patients by preventing chronic intracostal neuralgia and paraesthesia. However, accumulation of much more patient data is necessary to evaluate the clinical benefits of this method. Although we have chosen to refine MVATS in this report, we do not dismiss uniportal or subxiphoid lobectomy.

### Disclosure Statement

Appropriate written informed consent was obtained for publication of this case report and accompanying images.

## Supporting information


**Video S1.** Digest of operative movie. The operative method is the same as for conventional multiportal video‐assisted thoracic surgery for anatomical lobectomy.

**Video S2.** The video shows how to make a subxiphoid incision tunnelling into the right thoracic cavity for removal of the specimen.
